# Effects of *in vitro* Brevetoxin Exposure on Apoptosis and Cellular Metabolism in a Leukemic T Cell Line (Jurkat)

**DOI:** 10.3390/md20080014

**Published:** 2008-06-10

**Authors:** Catherine J. Walsh, Stephanie R. Leggett, Kathryn Strohbehn, Richard H. Pierce, John W. Sleasman

**Affiliations:** 1 Marine Immunology Program, Center for Shark Research, Mote Marine Laboratory, Sarasota, FL 34236, USA; 2 Chemical Fate and Effects Program, Center for Ecotoxicology, Mote Marine Laboratory, Sarasota, FL 34236, USA; 3 Division of Allergy, Immunology, and Rheumatology, Department of Pediatrics, University of South Florida, All Children’s Hospital, St. Petersburg, FL 33701, USA

**Keywords:** red tide, brevetoxin, Karenia brevis, immunotoxicity

## Abstract

Harmful algal blooms (HABs) of the toxic dinoflagellate, *Karenia brevis*, produce red tide toxins, or brevetoxins. Significant health effects associated with red tide toxin exposure have been reported in sea life and in humans, with brevetoxins documented within immune cells from many species. The objective of this research was to investigate potential immunotoxic effects of brevetoxins using a leukemic T cell line (Jurkat) as an *in vitro* model system. Viability, cell proliferation, and apoptosis assays were conducted using brevetoxin congeners PbTx-2, PbTx-3, and PbTx-6. The effects of *in vitro* brevetoxin exposure on cell viability and cellular metabolism or proliferation were determined using trypan blue and MTT (1-(4,5-dimethylthiazol-2-yl)-3,5-diphenylformazan), respectively. Using MTT, cellular metabolic activity was decreased in Jurkat cells exposed to 5 – 10 μg/ml PbTx-2 or PbTx-6. After 3 h, no significant effects on cell viability were observed with any toxin congener in concentrations up to 10 μg/ml. Viability decreased dramatically after 24 h in cells treated with PbTx-2 or -6. Apoptosis, as measured by caspase-3 activity, was significantly increased in cells exposed to PbTx-2 or PbTx-6. In summary, brevetoxin congeners varied in effects on Jurkat cells, with PbTx-2 and PbTx-6 eliciting greater cellular effects compared to PbTx-3.

## 1. Introduction

Harmful algal blooms (HABs) occur worldwide and have been identified as one of the key threats to human health [1–4]. Off the southwestern coast of Florida, blooms of the toxic dinoflagellate, *Karenia brevis*, have occurred almost annually in the Gulf of Mexico for the last twenty-five years. *K. brevis* blooms, known to the public as “red tide”, produce a suite of polyether neurotoxins, collectively termed brevetoxins, that result in massive fish kills, large numbers of mortalities in sea turtles and marine mammals, contamination of shellfish, and severe respiratory effects in humans.

Florida red tide is known to affect human health through two routes: neurotoxic shellfish poisoning (NSP) and inhalation. NSP results from consumption of contaminated shellfish, but has been well controlled through on-going shellfish monitoring programs. Inhalation of aerosolized toxins is also a route for human exposure, as the toxins produced by *K. brevis* blooms can be incorporated into marine aerosol [5]. Several brevetoxin congeners are produced by *K. brevis*, with brevetoxin-2 (PbTx-2) and brevetoxin-3 (PbTx-3) the most predominant forms isolated from seawater and marine aerosols [5,6]. Other toxin congeners, including PbTx-1, -6, and -9 and several new brevetoxin derivatives have been identified in natural blooms and cultures of *K. brevis* [7,8]. In humans, inhalation of aerosolized toxin produces a dry choking cough, stinging eyes and nose, and can trigger asthma attacks and exacerbate chronic obstructive pulmonary disease. Field studies indicate that humans with healthy as well as compromised airways respond to a red tide event [9–13].

Recent studies have suggested that airborne brevetoxins produced by red tide may be more harmful than health officials previously thought, with response to Florida red tide toxins possibly extending beyond initial acute reactions [13–15]. In support of this hypothesis are reports of a significant increase (54%) in emergency room admissions due to respiratory illnesses such as bronchitis and pneumonia, during a time period in which there was red tide compared to a time period without significant red tide in the area [11,14]. Such observations may be indicative of immunosuppression resulting from chronic exposure to aerosolized brevetoxins, and may be particularly important among persons with underlying respiratory disease [11,14]. Despite these facts, our current understanding of possible systemic health effects associated with aerosolized brevetoxins beyond initial respiratory effects is limited.

Animal models indicate that brevetoxin exposure has the potential to affect the immune system in many species, including manatee [16,17], cormorant [18], and rat [19–21]. Demonstrated immune functional impairment resulting from brevetoxin exposure includes reduced phagocytosis [19], decreased plaque-forming ability [19,20], and decreased lymphocyte proliferation [17]. *In vitro* brevetoxin exposure has been reported to result in DNA damage in human lymphocytes [22] and to affect the growth of cell lines [23,24].

The long-term effects associated with brevetoxin exposure are unknown and evidence is accumulating that brevetoxins may compromise health. Characterization of cellular consequences of brevetoxin exposure in the immune system is critical to fully understand the impact of recurrent red tide events on human health. The purpose of this investigation was to further current understanding of the effects of brevetoxins on immune cells. Using a Jurkat cell line, cell viability, cellular metabolism, and apoptosis were investigated following *in vitro* incubation with brevetoxins.

## 2. Results and Discussion

### 2.1 Effects of toxin on cell viability

Experiments were conducted to determine effects of toxin congeners PbTx-2, -3, and -6 on cell viability using a Jurkat cell line as target cells. The Jurkat cell line is a transformed cell line derived from human T-lymphocytes and is widely used to investigate a variety of cellular effects and immune system responses, due to the fact that these cells have many features in common with primary human T lymphocytes, including T-cell receptor, ion channels, and essential signaling pathways [25–27]. The results presented here represent *in vitro* data using this cell line, and as such may not necessarily be directly extrapolated to human immune system effects. Nonetheless, these data are valuable in providing initial mechanistic data and support for conducting additional research to determine the relevance of these data to human health.

**Figure 1:**
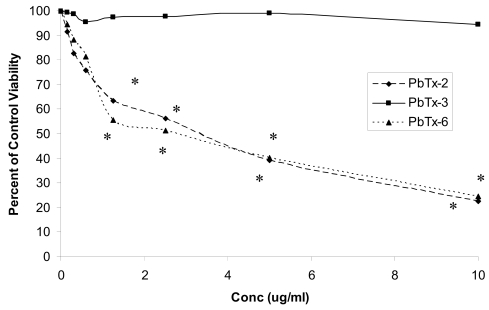
Percent viability of Jurkat cells treated with PbTx-2, PbTx-3, or PbTx-6 for 24 h. Data are represented as percent of ethanol control viability. Cells treated with PbTx-2 or PbTx-6 had significantly lower viability compared to corresponding ethanol controls and compared with viability of cells treated with PbTx-3 at concentrations indicated by *. Statistical differences compared to ethanol controls were determined using a *t*-test at each toxin concentration. Comparisons in viabilities among toxin congeners were determined using one-way ANOVA at each concentration. *N*=4.

With short-term incubation (3 h), cell viability did not decrease in response to treatment with toxins in concentrations as high as 10 μg/ml, with viability greater than 94% after all treatments. Cell viabilities following incubation of Jurkat cells with PbTx-2, -3, or -6 in concentrations of 0 – 10 μg/ml for 24 h are shown in Figure 1. Viabilities of cells treated with PbTx-2 or PbTx-6, but not with PbTx-3, were significantly lower (*P* < 0.05) than ethanol controls at toxin concentrations of 1.25 μg per ml or greater. Viabilities of cells treated with either PbTx-2 or -6 were also significantly reduced compared to viability of cells treated with PbTx-3 at concentrations of 1.25 μg/ml or greater. Viability of cells treated with PbTx-3 was above 95% of ethanol control at all concentrations after 24 h. Statistical differences compared to ethanol controls were determined using a *t*-test at each toxin concentration. Comparisons in viabilities among toxin congeners were determined using one-way ANOVA at each concentration. After 48 h, viability was extremely low in cells treated with PbTx-2 or -6, with viabilities less than 30% in response to all toxin concentrations. Of interest is the observation that Jurkat cells treated with PbTx-3 maintained viabilities greater than 95% of ethanol control over the 48 h incubation period at all toxin concentrations. Viabilities of ethanol control cells over the 48 h incubation period did not differ from cells treated with media only and were greater than 95% after 24 h and greater than 92% after 48 h.

### 2.2 Effects of brevetoxins on cellular metabolism measured using MTT

**Figure 2:**
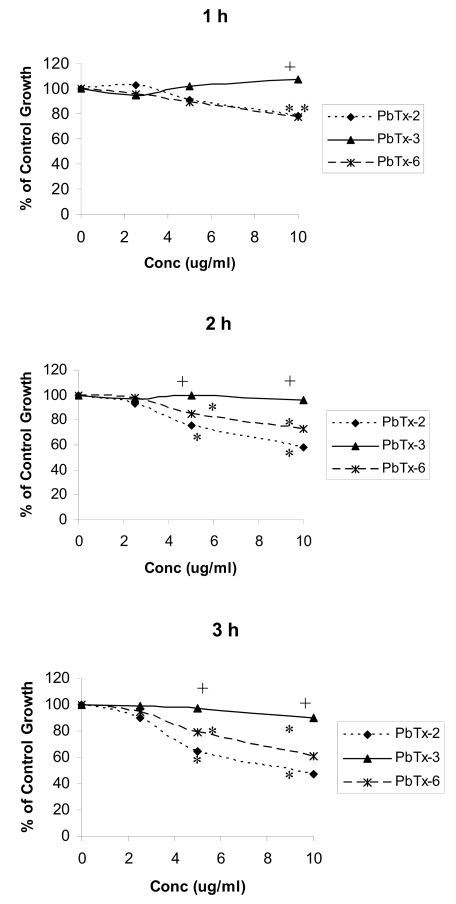
Effect of PbTx-2, PbTx-3, and PbTx-6 on cell metabolic activity in Jurkat cells exposed to 0 – 10 μg/ml toxin for 1, 2, or 3 h. Cellular metabolic activity was measured using MTT and reported as percentage of ethanol control. Cells treated with PbTx-2 and -6 had significantly lower metabolic activity compared with corresponding ethanol controls at 5 and 10 μg/ml, as indicated by the *. Cells treated with PbTx-2 or PbTx-6 had significantly lower metabolic activity compared to cells treated with PbTx-3 at 5 and 10 μg/ml, as indicated by the +. *Significantly less (*P* < 0.05) than ethanol control. +Significantly less (*P* < 0.05) than treatment with PbTx-3. Changes in cellular metabolic activity were compared to ethanol control for each toxin congener at each concentration using a *t*-test. Changes in cellular metabolic activity in cells treated with different toxin congeners were determined using one-way ANOVA at each toxin concentration. *N*=4.

The metabolic activity of brevetoxin-treated Jurkat cells was used as an indication of cell proliferation and was measured using MTT. Effects of brevetoxins on cellular metabolic activity in Jurkat cells after 1 – 3 h are shown in Figure 2 and are reported as percentage of ethanol control. Treatment of Jurkat cells with PbTx-2 or -6 resulted in greater effects on cell metabolic activity with increasing toxin concentration and exposure time. After 1, 2, and 3 h incubations, PbTx-2 and PbTx-6 significantly (*P* < 0.05) decreased cellular metabolic activity at concentrations of 5 and 10 μg/ml. After 3 h brevetoxin treatment, metabolic activity observed in response to 5 and 10 μg/ml PbTx-2 was 64 and 47% of ethanol control, respectively. In response to treatment with PbTx-6, metabolic activity was 80% and 60% of ethanol control in response to treatment with 5 and 10 μg/ml, respectively. During these time periods, PbTx-3 did not significantly affect cellular metabolism. Cells treated with PbTx-2 or PbTx-6 had significantly lower metabolic activity compared to cells treated with PbTx-3 at 10 μg/ml after 1, 2, and 3 h incubations and at 5 μg/ml after 2 and 3 h incubations.

**Figure 3:**
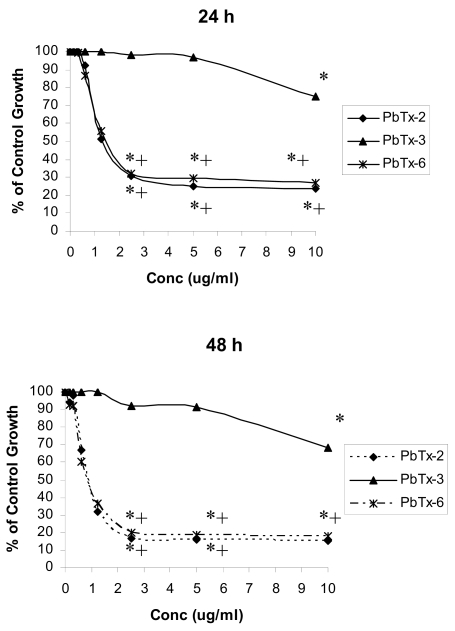
Effect of PbTx-2, PbTx-3, and PbTx-6 on cell metabolic activity in Jurkat cells exposed to 0 – 10 μg/ml toxin for 24 or 48 h. Cellular metabolic activity was measured using MTT and reported as a percentage of ethanol control. Cells treated with brevetoxins had significantly lower metabolic activity compared with corresponding ethanol controls at concentrations indicated by *. Cells treated with PbTx-2 or PbTx-6 had significantly less metabolic activity compared to cells treated with PbTx-3 at concentrations indicated by +. *Significantly less (*P* < 0.05) than ethanol control. +Significantly less (*P* < 0.05) than treatment with PbTx-3. Changes in cellular metabolic activity were compared to ethanol control for each toxin congener at each concentration using a *t*-test. Changes in cellular metabolic activity in cells treated with different toxin congeners were determined using one-way ANOVA at each toxin concentration. *N*=3.

Cell metabolic activity in cells treated with brevetoxins for 24 or 48 h is shown in Figure 3. Control cellular metabolic activity following exposure to PbTx-2 and PbTx-6 were significantly decreased (*P* < 0.05) compared to ethanol control at concentrations of 2.5 μg/ml or greater after both 24 and 48 h incubation periods. At toxin (PbTx-2 and PbTx-6) concentrations of 1.25 μg/ml, cells demonstrated approximately 50% of ethanol control metabolic activity after 24 h, but this decrease was not statistically significant. At concentrations of 2.5 μg/ml or greater, cells treated with PbTx-2 or PbTx-6 had significantly less cellular metabolic activity compared with cells treated with PbTx-3 at each concentration. Cells treated with PbTx-3 demonstrated significantly (*P* < 0.05) reduced metabolic activity compared to ethanol control in response to 10 μg/ml toxin, but not at lower toxin concentrations, after both 24 and 48 h incubations, with responses approximately 70–75% that of ethanol controls at the same concentration.

### 2.3 Effects of in vitro brevetoxin exposure on apoptosis in Jurkat cells

**Figure 4:**
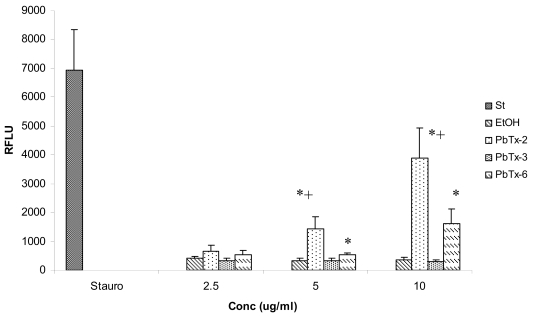
Caspase-3 activity in Jurkat cells incubated with 0 – 10 μg/ml PbTx-2, PbTx-3, or PbTx-6 for 3 h. St = Staurosporine; EtOH = ethanol control. *N* =5. Cells treated with PbTx-2 or PbTx-6 had significantly greater (*P* < 0.05) caspase-3 activity compared with corresponding ethanol controls at the concentrations indicated by *, as determined using a *t*-test at each concentration. Cells treated with PbTx-2 had significantly greater (*P* < 0.05) caspase-3 activity compared to cells treated with PbTx-3 at concentrations indicated by +, as determined using a one-way ANOVA at each concentration.

The effects of exposure to PbTx-2, PbTx-3, and PbTx-6 on apoptosis induction were determined by caspase-3 activity using a fluorescent microplate assay (Figure 4). Caspase-3 activity was measured after 3 h in response to treatment with 0–10 μg/ml toxin concentrations. Using a *t*-test to compare apoptotic activity in toxin treated cells with apoptotic activity in cells treated with ethanol control, cells treated with PbTx-2 or PbTx-6 were found to have significantly greater (*P* < 0.05) caspase-3 activity compared to ethanol control after 3 h exposure at concentrations of 5 and10 μg/ml, but not at 2.5 μg/ml, with caspase activity increasing from 3- to 8-fold, depending on toxin concentration. Caspase-3 activity induced by PbTx-2 was also significantly greater (*P* < 0.05) than that induced by PbTx-3 at 5 and 10 μg/ml concentrations. Although treatment with PbTx-6 increased caspase-3 activity compared to treatment with PbTx-3 approximately 2- or 5-fold in response to 5 and 10 μg/ml, respectively, these differences were not statistically significant.

**Figure 5:**
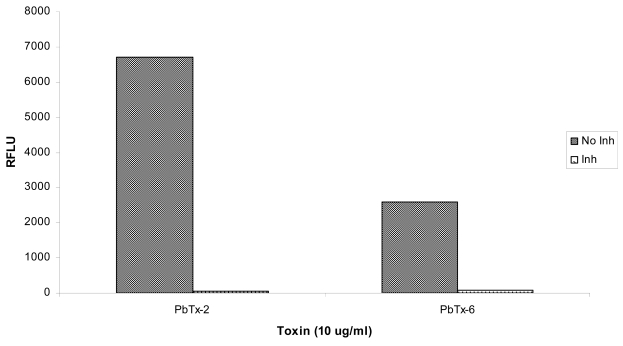
Effect of the caspase-3 inhibitor, Ac-DEVD-CHO, on caspase-3 activity in Jurkat cells exposed to 10 μg/ml PbTx-2 or PbTx-6 for 3 h without inhibitor (No Inh) or with 20 μM of Ac-DEVD-CHO (Inh). The data presented are an average of two trials of the experiment.

Jurkat cells exposed to brevetoxins *in vitro* were also treated with the caspase-3 inhibitor, Ac-DEVD-CHO. As shown in Figure 5, activation of caspase-3 in brevetoxin-exposed cells was reduced greater than 100-fold by treatment with this inhibitor. The results presented in Figure 5 represent an average of two experiments, therefore, no statistical analyses were conducted on these data.

Although several studies have led to the suggestion that respiratory effects associated with red tide toxin exposure may not represent the full impact of brevetoxins on human health, the effects of brevetoxins at the cellular level are not well understood, particularly in immune cells. The objective of the present study was to contribute to current understanding of potential effects of brevetoxin exposure on immune cells using *in vitro* treatments of a human leukemic T cell line, Jurkat cells, which have been widely used to approximate various cellular effects.

Although useful, it is difficult to make assumptions about cellular effects about environmental exposure based on studies with transformed cell lines such as the one used here. Although it is unlikely that results similar to the *in vitro* data presented here will be observed in immune cells following environmental exposure in humans, *in vitro* investigations such as these do play an important role in understanding human health risk assessment by providing initial mechanistic data [28], albeit without the pathways of absorption, distribution, and excretion that would be involved following environmental exposure. Although *in vitro* data with a transformed cell line cannot be directly extrapolated to human health risks, these provide valuable information to help direct future investigations of environmental exposure to brevetoxins in humans.

Brevetoxins remain in blood for several hours to days post-exposure [29–31] and become widely distributed throughout tissues [19,32]. Blood lipoproteins bind brevetoxins [33,34], which increases systemic distribution as well as the ability of toxin to enter cells. Placental transport of PbTx-3 also occurs [35]. These observations lend support to the hypothesis that brevetoxin exposure has the capacity to impact the immune system in that blood is continuously in contact with tissues and is host to effector immune cells.

Several potential mechanisms of action on immune system components have been suggested, including inhibition of cathepsin active sites [36,37], induction of apoptosis [16,22], and release of inflammatory mediators [16]. Effects on the cell cycle have also been hypothesized, based on reports of reduced lymphocyte proliferation in manatees [17], aberrant cell division in a cell line (SP2/0) exposed to brevetoxin *in vitro* [23], and inhibition of cellular proliferation of CHO-K1-BH4 cells [24]. In this paper, we address effects on brevetoxin on cellular metabolic activity and apoptosis.

Previously published reports on effects of brevetoxin on cell proliferation have included use of a reagent based on mitochondrial dehydrogenase activity, WST-1 [23], and determination of cell number [24]. MTT, a measure of metabolic activity, specifically mitochondrial function, of viable cells, has been used as a measure of cell proliferation [38–40] in other systems. Since proliferating cells are metabolically more active than non-proliferating cells, these assays have been considered suitable for measuring cell activation and proliferation [38]. In the present study, we report results on effects of brevetoxins using MTT as an indicator of metabolic activity, or proliferation, in a cell line. Jurkat cells treated with brevetoxin demonstrated significantly lower cellular metabolic activity, compared to control cells. Depending on toxin congener used, concentrations as low as 2.5 μg/ml significantly decreased this response compared to control. Han et al [23] reported a transient increase in cellular activity after 1.5 h exposure to brevetoxin, an effect which was not observed in the present study. Han et al used crude toxin extract at low concentrations, which may account for the observed differences. Using an increase in cell number as a measure, Sayer et al [24] reported decreased cell proliferation after 48 h treatment in response to toxin concentrations as low as 10^−12^ M. Different cell types were used in both of these studies, and it is highly likely that cell types may vary in their responses to brevetoxin treatment. Of interest is the observation that treatment with PbTx-3 did not significantly decrease cellular activity or viability until a high toxin concentration (10 μg/ml). These results indicate that the brevetoxin congener, PbTx-3, is considerably less cytotoxic towards Jurkat cells than are congeners PbTx-2 or -6.

Although many methods can be used to detect apoptosis, activation of caspase-3 is among the more commonly used. In the present study, caspase-3 activity was used to measure apoptosis in Jurkat cells exposed to brevetoxins *in vitro*, with a significant increase in apoptotic signaling events in response to PbTx-2 and -6, but not PbTx-3, observed. Although high concentrations of toxin were required to elicit activation of caspase-3, these data suggest that PbTx-3 and -6 have the ability to induce apoptosis, observations which are consistent with DNA damage reported by Sayer et al [22] and activation of interleukin-1-converting enzyme observed in manatees [16]. Sayer et al [22] reported DNA damage occurring in response to much lower brevetoxin concentrations, approximately 800 nM, with no differences among toxins.

Apoptosis primarily occurs through two defined pathways: the death receptor, or extrinsic, and the mitochondrial, or intrinsic, pathways. The extrinsic pathway, initiated by a family of cell surface death receptors, generates an apoptotic signal which may not involve mitochondria. The intrinsic pathway, on the other hand, is triggered by various signals which lead to disruption of mitochondrial function and activation of caspases, including caspase-3. Activation of caspases is responsible for events leading to morphological changes associated with apoptosis, such as DNA degradation, chromatin condensation, and membrane blebbing. The intrinsic pathway is regulated by the Bcl-2 family of proteins which includes Bax/Bak as integral components. The intrinsic pathway of apoptosis also involves disruption of mitochondrial function by affecting the transmembrane potential across the mitochondrial membrane and leading to the release of cytochrome c. The results reported here in which a decrease in cell metabolic activity, i.e., mitochondrial function, was demonstrated in immune cells exposed to brevetoxins *in vitro*, lends support to the hypothesis that brevetoxins may induce apoptosis through the intrinsic pathway. Based on activation of caspase-3 by brevetoxins, reports of DNA damage, and elimination of caspase-3 activity using the inhibitor Ac-DEVD-CHO, it is likely that brevetoxin-induced apoptosis proceeds through the intrinsic pathway and involves critical mediators such as Bax/Bak. Although apoptosis does occur in response to brevetoxin treatment, due to the high toxin concentrations required, the results of the present study do not support the hypothesis that apoptosis is a primary mechanism of toxin effect in an immune-derived transformed cell line.

The data presented here consistently indicate that PbTx-3 exerts fewer effects on Jurkat cells compared with PbTx-2 or -6. Reports of effects of different toxin congeners vary, with some studies reporting no measurable differences in cellular effects elicited by toxin congeners either *in vitro* [22] or *in vivo* [41], whereas other studies do report congener-dependent effects [24,29]. Specifically, Radwan et al [29] report different processing of PbTx-2 and -3 in an animal model. Some differences in cellular effects elicited toxin congeners may be attributed to specific receptors, cellular metabolism, or biotransformation enzymes in target cells. Certain structural features among brevetoxins contribute to varying potencies and mechanisms of action [38–40]. For example, due to the very reactive α,β-unsaturated aldehyde group of PbTx-2, this congener is more reactive toward glutathione compared to PbTx-3, and is rapidly metabolized and excreted [29]. PbTx-6, but not -2 or -3, binds the aryl hydrocarbon receptor [45]. PbTx-6 is an epoxide metabolite and readily binds cellular molecules, which has the potential to lead to genotoxic effects [46].

Brevetoxins have been shown to undergo cellular metabolism through known xenobiotic biotransformation pathways for detoxification, primarily in the liver [25,45–47]. Although liver is considered the primary site for xenobiotic transformation, many of these enzymes are also present in peripheral blood lymphocytes and function in toxin metabolism [48–50]. The presence of these enzymes in immune cells may contribute to some of the variation in cytotoxic effects of toxin congeners observed in the research presented here. Brevetoxin metabolites generated by biotransformation pathways in immune cells may result in potentially significant and long-lasting impacts on the immune system.

*Karenia brevis* red tide blooms along the Florida Gulf Coast generally result in brevetoxin concentrations in seawater from 5 – 30 μg/L, depending on many factors, including cell count and age of the bloom [5], with variations in the toxin congener composition of each bloom. The experiments conducted in this study utilized brevetoxin concentrations that were much higher than environmental exposure, an approach not uncommon for initial toxicology studies, in order to direct future research geared towards understanding effects of environmental exposures. In that regard, high toxin concentrations were utilized in short-term *in vitro* experiments to facilitate detection of cellular events potentially impacted by brevetoxin exposure. Although toxin concentrations used in these *in vitro* studies are not likely to be encountered in the environment in a single exposure event, circulation and bioaccumulation of toxins may result in substantially increased toxin load over repeat exposures.

The objective of this study was to determine how brevetoxins, naturally produced in the marine environment, may potentially affect cells of immune origin. Harmful effects of brevetoxins on the health of sealife have been well-documented [16–18]. Effects on human health, beyond respiratory irritation, are essentially unknown, with immunotoxic effects of brevetoxins only in the initial phases of investigation. The range of *K. brevis* blooms is extending [1], potentially impacting a greater percentage of the population. Also of concern is the fact that brevetoxins can become aerosolized and carried greater than a mile inland, thus increasing the range of potential brevetoxin exposure beyond the immediate coastline (Kirkpatrick *et al.*, pers comm.). Although exposure to harmful algal blooms is a significant health concern, few reports have been published regarding immune system effects associated with brevetoxin exposure. To improve our understanding of potential effects of red tide toxin on the immune system, *in vitro* experiments using a leukemic T cell line, Jurkat, were conducted. The results presented here suggest brevetoxin exposure may elicit important cellular effects, including apoptosis, cell metabolism and proliferation, and cytotoxicity, in an immune-derived transformed cell line following *in vitro* exposure. These observations support the hypothesis that brevetoxin exposure may affect cellular functions and consequently, may have the potential to impact immune function, although additional studies are required to determine the relevance of these data to human health. Of interest is the fact that one of the major toxin congeners detected in marine aerosols [6], PbTx-3, demonstrated only minimal effects on immune cells, although these effects became enhanced with increasing time of exposure and concentration. On the other hand, PbTx-2, another major toxin congener in *K. brevis* blooms [6], appears to disrupt cellular metabolism and induce apoptosis in immune cell targets in this *in vitro* cell culture system. PbTx-6, a minor and infrequent component of blooms [6] and a biotransformation product of PbTx-2 [46], elicits cellular effects similar to those observed with PbTx-2. The data presented here generate additional questions regarding effects of brevetoxins on immune cells at the cellular level and indicate that such effects warrant further in-depth investigations into mechanisms of action of brevetoxins on immune cells. Exposure to aerosolized harmful algal blooms may be a significant health concern and characterization of effects of red tide toxins on immune health is essential. *In vitro* experiments such as these provide direction for future research efforts to more fully understand the pathogenesis of brevetoxin exposure at the cellular level and help delineate potential harmful effects of brevetoxins on immune function.

## 3. Experimental Section

### 3.1 Cells

The human leukemic T cell line, Jurkat clone E-6, was obtained from the American Type Culture Collection (Manassas, VA). Cells were cultured in RPMI 1640, pH 7.4, containing 10% heat-inactivated fetal bovine serum (FBS; Hyclone, Logan UT), 100 units/ml penicillin-streptomycin (Sigma Chemical Co., St. Louis, MO), and 100 μg/ml amphotericin B (Sigma). Cells were cultured in a humidified 5% CO_2_ atmosphere at 37 ºC and media renewed every 2–3 days as recommended.

### 3.2 Toxins

Brevetoxin congeners PbTx-2, PbTx-3, and PbTx-6 were obtained from Dr. Dan Baden, University of North Carolina, Wilmington, NC. Individual neat toxin standards were reconstituted at a concentration of 500 μg/ml in a solution of 80% ethanol in HPLC water. To confirm purity and toxin concentration, brevetoxin analyses were performed by liquid chromatography mass spectrometry (LC/MS) using a ThermoFinnigan AqA LC/MS (Thermo Electron Corp, Madison, WI) single quad system scanned from 204–1216 AMU according to the procedures of Pierce *et al.* [51]. The column was a Phenomenex Luna C-18 reverse-phase 5 μ particle size, 250 mm x 2 mm analytical column with solvent gradient of acidified (0.3% acetic acid) acetonitrile (ACN/H_2_O) with initial 50:50 ACN/H_2_O over 40 min. The instrument was calibrated with a standard brevetoxin mixture containing PbTx-2 and PbTx-3. The toxin concentrations used in cell cultures ranged from 0.15 – 10 μg/ml (0.17 – 11 μM) final concentration. For each toxin concentration, an ethanol control was prepared from 80% ethanol in HPLC water used to reconstitute neat toxins.

### 3.3 Cell Viability

Effects of toxin exposure on cell viability were determined using trypan blue exclusion [50]. Cells were adjusted to a concentration of 2 x 10^6^ cells/ml, distributed into wells of a 96-well plate, and synchronized by overnight serum starvation. Plates were centrifuged at 300 x g for 7 min, the supernatant removed, and 100 μl cell culture medium (RPMI 1640 containing 10% FBS) and the appropriate concentration (0 – 10 μg/ml) of PbTx-2, PbTx-3, or PbTx-6 was added. After 3, 24, or 48 h incubation periods, the cells were diluted 1:1 with trypan blue and counted using a hemacytometer. The percentage of live cells in each treatment was determined. Statistical differences in cell viabilities were determined by comparing raw data for cell viabilities at each toxin concentration with the corresponding ethanol control using Student’s *t*-test, with *P* < 0.05 considered significant. Statistical differences in viabilities among toxin congeners were determined using a one-way ANOVA at each toxin concentration.

### 3.4 MTT Assay

For this study, metabolic activity of viable Jurkat cells was used as a measure of cell proliferation. Cellular metabolic activity was measured using an MTT (1-(4,5-dimethylthiazol-2-yl)-3,5-diphenylformazan) assay which is now widely used to quantitate cellular proliferation and cytotoxicity [38,53]. Since proliferating cells are metabolically more active than non-proliferating or resting cells, such tetrazolium salt-based assays are frequently used to measure cell activation and proliferation.

To synchronize the cells, Jurkat cells were incubated in serum-free medium overnight at 37 ºC in a 5% CO_2_ humidified atmosphere. Experiments were done in quadruplicate using separate cell cultures. After 24 h, cells were centrifuged, supernatant carefully removed and cells resuspended in cell culture medium (RPMI 1640 containing 10% FBS) containing brevetoxins in concentrations ranging from 0 – 10 μg/ml. Cultures were incubated for 1, 2, 3, 24, or 48 h time periods. Ethanol controls were included in all experiments. After the incubation period, 25 μl MTT from a 5 mg/ml stock solution prepared in PBS was added to each well. Plates were returned to the incubator for 4 h. After the 4 h incubation period, 100 μl of a solubilizing solution (0.1 N HCl, 10% SDS) was added and the plates incubated overnight at 37 ºC. MTT conversion was measured using a microplate reader (BioTek ELx800) with absorbance read at 570 nm and background absorbance at 630 nm subtracted. Cell proliferation or metabolism was expressed as percentage of ethanol control cells. Statistical analyses were conducted on raw data to compare cellular metabolic activity in brevetoxin-treated cells compared to ethanol control cells, with a *t*-test conducted with data from each individual toxin congener at each concentration compared to the corresponding ethanol control. Significant differences among toxin congeners at each concentration were examined using a one-way ANOVA to compare cellular metabolic activity among cells treated with PbTx-2, PbTx-3, or PbTx-6.

### 3.5 Caspase-3 activity

Apoptosis occurring in response to treatment with brevetoxin was measured using a caspase-3 fluorescent microplate assay (Molecular Probes, Eugene, OR). Jurkat cells were adjusted to a concentration of 2 x 10^6^ cells/ml and distributed in 100 μl volumes into wells of a 96-well microtiter plate and incubated with 0 – 10 μg/ml PbTx-2, PbTx-3, or PbTx-6. Staurosporine (2 μM) was used as a positive control. After 3 h incubation at 37 ºC, 5% CO_2_, cells were pelleted by centrifugation at 600 x g for 5 min and washed once with PBS. Cells were resuspended in 50 μl lysis buffer (20 mM Tris, pH 7.5, 0.2 M NaCl, 2 mM EDTA, 0.02% Triton X-100), lysed by incubating on ice for 30 min, and pelleted to remove debris. Supernatant was transferred from each sample to wells of a black microplate. Substrate (Z-DEVD-R110) was added with reaction buffer (10 mM PIPES, pH 7.4, 2 mM EDTA, 0.1% CHAPS) and incubated at room temperature in the dark for 30 min. In some wells, 20 μM Ac-DEVD-CHO, a potent inhibitor of caspase-3 [55], was added 10 min before adding substrate. Fluorescence was measured using a microplate reader (BioTek FLx800) with excitation at 496 nm and emission at 520 nm. Statistical comparisons were made between toxin treated cells and ethanol control at each concentration using a *t*-test. Statistical comparisons were also made among toxin congeners at each concentration using one-way ANOVA.

### 3.6 Statistical techniques

Statistical analyses were conducted using SigmaStat Version 3.11. Statistical comparisons were made using either the Student’s *t*-test or one way analysis of variance (ANOVA). Tests conducted for each experiment are listed in the section describing that experiment and in the figure legends. *P* values less than 0.05 were considered significant. Where included, data points and error bars on the figures represent mean ± standard error of the mean (SEM) responses.

## References

[1] Van Dolah FM (2000). Marine Algal Toxins: Origins, Health Effects, and Their Increased Occurrence. Environ Health Perspect.

[2] Knap A, Dwailly E, Furgal C, Galvin J, Baden D, Bowen RE, Depledge M, Duguay L, Fleming LE, Ford T, Moser F, Owen R, Suk WA, Unluata U (2002). Indicators of Ocean Health and Human Health: Developing a Research and Monitoring Framework. Environ Health Perspect.

[3] Sandifer PA, Holland AF, Rowles TK, Scott GI (2004). The Oceans and Human Health. Environ Health Perspect.

[4] Tyson FL, Rice DL, Dearry A (2004). Connecting the Oceans and Human Health. Environ Health Perspect.

[5] Pierce RH, Henry MS, Blum PC (2008). Brevetoxin Abundance and Composition during ECOHAB-Florida Field Monitoring Cruises in the Gulf of Mexico. Continent Shelf Res.

[6] Cheng YS, Zhou Y, Irvin CM, Pierce RH, Naar J, Backer LC, Fleming LE, Kirkpatrick B, Baden DG (2005). Characterization of Marine Aerosol for Assessment of Human Exposure to Brevetoxins. Environ Health Perspect.

[7] Bourdelais J, Jacobs HM, Wright JLC, Bigwarf PM, Baden DG (2005). A New Polyether Ladder Compound Produced by the Dinoflagellate, *Karenia brevis*. J Nat Prod.

[8] Abraham A, Plakas SM, Wang Z, Jester EL, El Said KR, Granade HR, Henry MS, Blum PC, Pierce RH, Dickey RW (2006). Characterization of Polar Brevetoxin Derivatives Isolated from *Karenia brevis* Cultures and Natural Blooms. Toxicon.

[9] Backer LC, Fleming LE, Rowan A, Cheng Y-S, Benson J, Pierce RH, Zaias J, Bean J, Bossart GD, Johnson D, Quimbo R, Baden DG (2003). Recreational Exposure to Aerosolized Brevetoxins during Florida Red Tide Events. Harmful Algae.

[10] Backer LC, Kirkpatrick B, Fleming LE, Cheng YS, Pierce R, Bean JA, Clark R, Johnson D, Wanner A, Tamer R, Zhou Y, Baden DG (2005). Occupational Exposure to Aerosolized Brevetoxins during Florida Red Tide Events: Effects on a Healthy Worker Population. Environ Health Perspect.

[11] Kirkpatrick B, Fleming LE, Squicciarini D, Backer LC, Clark R, Abraham W, Benson J, Cheng YS, Johnson D, Pierce R, Zaias J, Bossart GD, Baden DG (2004). Literature Review of Florida Red Tide: Implications for Human Health Effects. Harmful Algae.

[12] Fleming LE, Backer LC, Baden DG (2005). Overview of Aerosolized Florida Red Tide Toxins: Exposures and Effects. Environ Health Perspect.

[13] Fleming LE, Kirkpatrick B, Backer LC, Bean JA, Wanner A, Dalpra D, Tamer R, Zaias J, Cheng YS, Pierce R, Naar J, Abraham W, Clark R, Zhou Y, Henry MS, Johnson D, Van de Bogart G, Bossart GD, Harrington M, Baden DG (2005). Initial Evaluation of the Effects of Aerosolized Florida Red Tide Toxins (Brevetoxins) in Persons with Asthma. Environ Health Perspect.

[14] Fleming LE, Kirkpatrick B, Backer LC, Bean JA, Wanner A, Reich A, Zaias J, Cheng YS, Pierce R, Naar J, Abraham WM, Baden DG (2007). Aerosolized Red-Tide Toxins (Brevetoxins) and Asthma. Chest.

[15] Kirkpatrick B, Fleming LE, Backer LC, Bean JA, Tamer R, Kirkpatrick G, Kane T, Wanner A, Dalpra D, Reich A, Baden DG (2006). Environmental Exposures to Florida Red Tides: Effects on Emergency Room Respiratory Diagnoses Admissions. Harmful Algae.

[16] Bossart GD, Baden DG, Ewing R, Roberts B, Wright S (1998). Brevetoxicosis in Manatees (*Trichechus manatus latirostris*) from the 1996 Epizootic: Gross, Histopathologic, and Immunocytochemical Features. Toxicol Pathol.

[17] Walsh CJ, Luer CA, Noyes DR (2005). Effects of Environmental Stressors on Lymphocyte Proliferation in Florida Manatees, *Trichechus manatus latirostris*. Vet Immunol Immunopathol.

[18] Kreuder C, Mazet JAK, Bossart GD, Carpenter TE, Holyoak M, Elie MS, Wright SD (2002). Clinicopathologic Features of Suspected Brevetoxicosis in Double-Crested Cormorants (*Phalacrocorax auritus*) Along the Florida Gulf Coast. J Zoo Wildlife Med.

[19] Benson JM, Tischler DL, Baden DG (1999). Uptake, Tissue Distribution, and Excretion of Brevetoxin 3 Administered to Rats by Intratracheal Instillation. J Toxicol Environ Health A.

[20] Benson J, Hahn F, March T, McDonald J, Sopori M, Seagrave J, Gomez A, Bourdelais A, Naar J, Zaias J, Bossart G, Baden D (2004). Inhalation Toxicity of Brevetoxin 3 in Rats Exposed for 5 Days. J Toxicol Environ Health A.

[21] Benson JM, Hahn FF, March TH, McDonald JD, Gomez AP, Sopori MJ, Bourdelais AJ, Naar J, Zaias J, Bossart GD, Baden DG (2005). Inhalation Toxicity of Brevetoxin 3 in Rats Exposed for Twenty-two Days. Environ Health Perspect.

[22] Sayer A, Hu Q, Bourdelais AJ, Baden DG, Gibson JE (2005). The Effect of Brevetoxin-Induced DNA Damage in Human Lymphocytes. Genotoxicology.

[23] Han TK, Derby M, Martin DF, Wright SD, Dao ML (2002). Effects of Brevetoxins on Murine Myeloma SP2/O Cells. Aberrant Cellular Division. Int J Toxicol.

[24] Sayer A, Hu Q, Bourdelais AJ, Baden DG, Gibson JE (2006). The Inhibition of CHO-K1-BH4 Cell Proliferation and Induction of Chromosomal Aberrations by Brevetoxins *in vitro*. Food Chem Toxicol.

[25] Abraham RT, Weiss A (2004). Jurkat T cells and development of the T-cell receptor signaling paradigm. Nat Rev Immunol.

[26] Desai R, Peretz A, Idelson H, Lazarovici P, Attali B (2000). Ca^2+^ -activated K^+^ channels in human leukemic Jurkat T cells. J Biol Chem.

[27] Morimoto T, Ohya S, Hayashi H, Onozaki K, Imaizumi Y (2007). Cell-cycle-dependent regulation of Ca^2+^ -activated K^+^ channel in Jurkat T-Lymphocyte. J Pharmacol Sci.

[28] Eisenbrand G, Pool-Zobel B, Baker V, Balls M, Blaauboer BJ, Boobis A, Carere A, Kevekordes S, Pieters R, Kleiner J (2002). Methods of *in vitro* toxicology. Food Chem Toxicol.

[29] Radwan FF, Wang Z, Ramsdell JS (2005). Identification of a Rapid Detoxification Mechanism for Brevetoxin in Rats. Toxicol Sci.

[30] Woofter R, Dechraoui MY, Garthwaite I, Towers NR, Gordon CJ, Córdova J, Ramsdell JS (2003). Measurement of Brevetoxin Levels by Radioimmunoassay of Blood Collection Cards after Acute, Long-term, and Low Dose Exposure in Mice. Environ Health Perspect.

[31] Woofter RT, Brendtro K, Ramsdell JS (2005). Uptake and Elimination of Brevetoxin in Blood of Striped Mullet (*Mugil cephalus*) after Aqueous Exposure to *Karenia brevis*. Environ Health Perspect.

[32] Tibbetts BM, Baden DG, Benson JM (2006). Uptake, Tissue Distribution, and Excretion of Brevetoxin-3 Administered to Mice by Intratracheal Instillation. J Toxicol Environ Health A.

[33] Woofter RT, Spiess PC, Ramsdell JS (2005). Distribution of Brevetoxin (PbTx-3) in Mouse Plasma: Association with High-Density Lipoproteins. Environ Health Perspect.

[34] Woofter RT, Ramsdell JS (2007). Distribution of Brevetoxin to Lipoproteins in Human Plasma. Toxicon.

[35] Benson JM, Gomez AP, Statom GL, Tibbetts BM, Fleming LE, Backer LC, Reich A, Baden DG (2006). Placental Transport of Brevetoxin-3 in CD-1 Mice. Toxicon.

[36] Katunuma N, Matsunaga Y, Himeno K, Hayashi Y (2003). Insights into the Roles of Cathepsin in Antigen Processing and Presentation Revealed by Specific Inhibitors. Biol Chem.

[37] Sudarsanam S, Virca GD, March CJ, Srinivasan S (1992). An Approach to Computer-Aided Inhibitory Design: Application to Cathepsin L. J Comput Aided Mol Des.

[38] Goyarts T, Dänicke S, Tiemann U, Rothkötter HJ (2006). Effect of the Fusarium Toxin Deoxynivalenol (DON) on IgA, IgM, and IgG Concentrations and Proliferation of Porcine Blood Lymphocytes. Toxicol In vitro.

[39] Minervini F, Giannoccaro A, Cavallini A, Visconti A (2005). Investigations on Cellular Proliferation Induced by Zearalenone and Its Derivatives in Relation to the Estrogenic Parameters. Toxicol Lett.

[40] Khan KN, Masuzaki H, Fujishita A, Kitajima M, Kohno T, Sekine I, Matsuyama T, Ishimaru T (2005). Regulation of Hepatocyte Growth Factor by Basal and Stimulated Macrophages in Women with Endometriosis. Human Reprod.

[41] Abraham WM, Bourdelais AJ, Sabater JR, Ahmed A, Lee TA, Serebriakov I, Baden DG (2005). Airway Responses to Aerosolized Brevetoxin in an Animal Model of Asthma. Am J Respir Crit Care Med.

[42] Jeglitsch G, Rein K, Baden DG, Adams DJ (1998). Brevetoxin-3 (PbTx-3) and its Derivatives Modulate Single Tetrodotoxin-Sensitive Sodium Channels in Rat Sensory Neurons. J Pharmacol Exptl Therap.

[43] Purkerson SL, Baden DG, Fieber LA (1999). Brevetoxin Modulates Neuronal Sodium Channels in Two Cell Lines Derived from Rat Brain. Neurotoxicology.

[44] Baden DG, Bourdelais AJ, Jacocks H, Michelliza S, Naar J (2005). Natural and Derivative Brevetoxins: Historical Background, Multiplicity, and Effects. Environ Health Perspect.

[45] Washburn BS, Rein KS, Baden DG, Walsh PJ, Hinton DE, Tullis K, Denison MS (1997). Brevetoxin-6 (PbTx-6), a Nonaromatic Marine Neurotoxin, is a Ligand of the Aryl Hydrocarbon Receptor. Arch Biochem Biophys.

[46] Radwan FF, Ramsdell JS (2006). Characterization of *In vitro* Oxidative and Conjugative Metabolic Pathways for Brevetoxin (PbTx-2). Toxicol Sci.

[47] Washburn BS, Baden DG, Gassman NJ, Walsh PJ (1994). Brevetoxin: Tissue Distribution and Effect on Cytochrome P450 Enzymes in Fish. Toxicon.

[48] Dey A, Dhawan A, Kishore Seth P, Parma D (2005). Evidence for Cytochrome P450 2E1 Catalytic Activity and Expression in Rat Blood Lymphocytes. Life Sci.

[49] Haas CE, Brazeau D, Cloen D, Booker BM, Frerichs V, Zaranek C, Frye RF, Kufel T (2005). Cytochrome P450 mRNA Expression in Peripheral Blood Lymphocytes as a Predictor of Enzyme Induction. Eur J Clin Parmacol.

[50] Van Duursen MBM, Sanderson JT, Van den Berg M (2005). Cytochrome P450 1A1 and 1B1 in Human Blood Lymphocytes are Not Suitable as Biomarkers of Exposure to Dioxin-Like Compounds: Polymorphisms and Interindividual Variation in Expression and Inducibility. Toxicol Sci.

[51] Pierce RH, Henry MS, Blum PC, Hamel SL, Kirkpatrick B, Cheng YS, Zhou Y, Irvin CM, Naar J, Weidner A, Fleming LE, Backer LC, Baden DG (2005). Brevetoxin Composition in Water and Marine Aerosol Along a Florida Beach: Assessing Potential Human Exposure to Marine Biotoxins. Harmful Algae.

[52] Baur H, Kaspare S, Pfaff E (1975). Criteria of Viability of Isolated Liver Cells. Hoppe-Seylers Z Physiol Chem.

[53] Mosmann TJ (1983). Rapid Colorimetric Assay for Cellular Growth and Survival: Application to Proliferation and Cytotoxicity Assays. J Immunol Meth.

[54] Sugawara J, Fukaya T, Murakami T, Yoshida H, Yajima A (1997). Hepatocyte Growth Factor Stimulates Proliferation, Migration, and Lumen Formation of Human Endometrial Epithelial Cells *in vitro*. Biol Reprod.

[55] Nicholson DW, Ali A, Thornberry NA, Vaillancourt JP, Ding CK, Gallant M, Gareau Y, Griffin PR, Labelle M, Lazebnik YA (1995). Identification and inhibition of the ICE/CED-3 protease necessary for mammalian apoptosis. Nature.

